# Community access to palliative care medicines – patient and professional experience: systematic review and narrative synthesis

**DOI:** 10.1136/bmjspcare-2020-002761

**Published:** 2021-03-28

**Authors:** Mizue Ogi, Natasha Campling, Jakki Birtwistle, Alison Richardson, Michael I Bennett, Miriam Santer, Susan Latter

**Affiliations:** 1 School of Health Sciences, University of Southampton, Southampton, Hampshire, UK; 2 Leeds Institute of Health Sciences, University of Leeds, Leeds, UK; 3 University Hospital Southampton NHS Foundation Trust, Southampton, UK; 4 Leeds Institute of Health Sciences, University of Leeds, Leeds, West Yorkshire, UK; 5 School of Primary Care, Population Sciences and Medical Education, University of Southampton, Southampton, Hampshire, UK

**Keywords:** home care, service evaluation, symptoms and symptom management

## Abstract

**Background:**

Providing palliative care patients living at home with timely access to medicines is critical to enable effective symptom management, minimise burden and reduce unplanned use of healthcare services. Little is known about how diverse community-based palliative care models influence medicine access.

**Objective:**

To produce a critical overview of research on experiences and outcomes of medicine access in community-based palliative care models of service delivery through a systematic review and narrative synthesis.

**Methods:**

MEDLINE, CINAHL, EMBASE, PsycINFO, Cochrane Library databases and grey literature were systematically searched for all types of studies. Study quality was assessed using the Mixed Methods Appraisal Tool; a narrative synthesis was used to integrate and summarise findings.

**Results:**

3331 articles were screened; 10 studies were included in the final sample. Studies included a focus on community pharmacy (n=4), hospice emergency medication kits (HEMKs) in the home (n=3), specialist community nurse prescribers (n=1), general practice (n=1) and one study included multiple service delivery components. Community pharmacy was characterised by access delays due to lack of availability of medicine stock and communication difficulties between the pharmacy and other healthcare professionals. HEMKs were perceived to reduce medicine access time out of hours and speed symptom control. However, the majority of studies comprised small, local samples, largely limited to self-reports of health professionals. There was a lack of data on outcomes, and no comparisons between service delivery models.

**Conclusions:**

Further research is required to understand which models facilitate rapid and efficient access to medicines for community-based palliative care patients.

**Key messages**

**What was already known?**

- Access to medicines in the home in the last year of life is critical for symptom control and reducing distress.
- Patients and carers may experience problems in access, and processes can be lengthy and burdensome.
- A number of different models of end-of-life service delivery are in operation, including recent innovations.
- Little is known about how accessing medicines is experienced within service delivery models.

**What are the new findings?**

- Community pharmacy was characterised by delays in accessing medicines.
- Hospice-provided medication kits stored at home were perceived to reduce medicines’ access time out- of- hours, and speed symptom control.
- Few large scale, rigorous studies exist to allow conclusions about access experiences to be drawn.

**What is their significance?**

- Further research into experiences and outcomes of medicines access at end-of-life in the community is required

##  Introduction

 Population ageing, together with the home being many people’s preferred place of death,^1^ has increased the need for community-based palliative care, including access to medicines. Timely patient access to medicines in the last year of life (end of life (EoL)) is critical for control of symptoms managed at home.^2^ Patients may have a complex range of clinical issues related to their condition, and fluctuating symptoms, including severe pain, can be difficult to control, requiring frequent readjustment of medication.^3^ The process of medicine access for symptom control can be a lengthy one, including obtaining a prescription for a medicine, ensuring it is dispensed correctly and supplied, together with information that enables patients and carers to manage medicines effectively in the home. Evidence from our previous studies suggests that for patients and carers receiving community-based palliative care, the experience of accessing medicines is often a considerable burden, involving multiple professionals and including a complex process of attaining and managing controlled drugs, such as opioids.^4^

In the UK, the provision of community palliative care is characterised by heterogeneous models of service delivery.^5^ Care can be delivered by generalist or specialist health professionals, or a mix of both; patients may or may not receive care from specialist palliative care nurses, and these or other generalist nurses may or may not be trained to independently prescribe medicines directly to patients. Nurses and pharmacists in the UK have among the most extensive prescribing rights in the world, including the prescription of controlled drugs. The introduction of these prescribing rights was driven by the need to increase speed of access to medicines for patients and make best use of health professionals’ skills.^6^ Additionally, in some areas, community pharmacies may hold enhanced stocks of palliative care medicines and extended opening hours as part of specially commissioned services. Other more recent initiatives in service delivery may also impact the experience of medicine access: a policy drive to increase the availability of out-of-hours telephone advice for palliative care patients; and pharmacists with a prescribing qualification are increasingly employed in general practices in England in a move to diversify primary care workforce skill mix.^7^ Paramedics were also granted prescribing authority in the UK in 2019, who may be a first point of call for patients in an emergency.

However, little is known about the impact of these models of service delivery on patients’ experience of accessing EoL medicines in the community or the relative merits of each approach. Questions such as which models work best, do recent initiatives improve speed and lessen the burden on patients working to access medicines and what factors support or challenge access experiences remain unanswered.

The aim of the systematic review reported here was to produce a critical overview of existing research on experiences and outcomes of medicine access associated with models of service delivery of community-based EoL care. Our aim was to systematically search for and review international research studies for evidence of: (A) patient, carer and health professional experience of medicine access within the context of different models of service delivery, and (B) patient and carer outcomes associated with medicine access experiences. In this review, medicine access was defined as prescribing, dispensing, supplying and associated information provision about medicines.

This systematic review formed part of a larger study entitled ‘Accessing medicines at end-of-life: a multi-stakeholder, mixed-method evaluation of service provision’ (ACcess To MEDicines (ActMed) study; National Institute for Health Research-funded project (HS & DR 16/52/23)) (https://www.journalslibrary.nihr.ac.uk/programmes/hsdr/165223/#/).

## Methods

The systematic review followed the Preferred Reporting Items for Systematic Reviews and Meta-Analyses (PRISMA) reporting guidelines.^8^ The review is registered on the International Prospective Register of Systematic Reviews (PROSPERO) database (Ref No CRD42017083563) (http://www.crd.york.ac.uk/PROSPERO/display_record.php?ID=CRD42017083563).

### Search strategy

A search strategy was developed based on the research question, ‘What are the experiences and outcomes of medicine access for patients and carers receiving community-based models of palliative care, during the last year of life?’ The search was conducted as described below to include any studies related to this focus.

#### Information sources

1. Database search: Four electronic databases (MEDLINE, PsycINFO, CINAHL and EMBASE) were searched for published literature from January 2006 to March 2019 using keywords, synonyms and Boolean operators. An example of the search strategy is shown in online supplemental table 1.
2. Citation search: A citation search was conducted on the included articles for further relevant material.
3. Databases of systematic reviews: The Cochrane Library database was searched in order to acquire relevant systematic reviews published between January 2006 and June 2019.
4. Ongoing systematic reviews: To obtain information on relevant ongoing systematic reviews, PROSPERO was searched for reviews registered between January 2006 and June 2019.
5. Relevant research in progress: To gain information regarding relevant ongoing trials, ISRCTN and ClinicalTrials.gov were searched for trials registered between January 2006 and June 2019.
6. Experts’ lists: To obtain further relevant published or grey literature, members of the Scientific Steering Committee of the ActMed study were asked for their top five references on this issue.
7. Grey literature: To minimise the impact of publication bias, grey literature sources (British Library, King’s Fund, Networked Digital Library of Theses and Dissertation (NDLTD), National Institute for Health and Care Excellence Evidence Search, Nuffield Trust, OpenGrey and Google) were searched for unpublished materials between January 2006 and June 2019.

### Study selection

#### Eligibility

**Figure 1:**
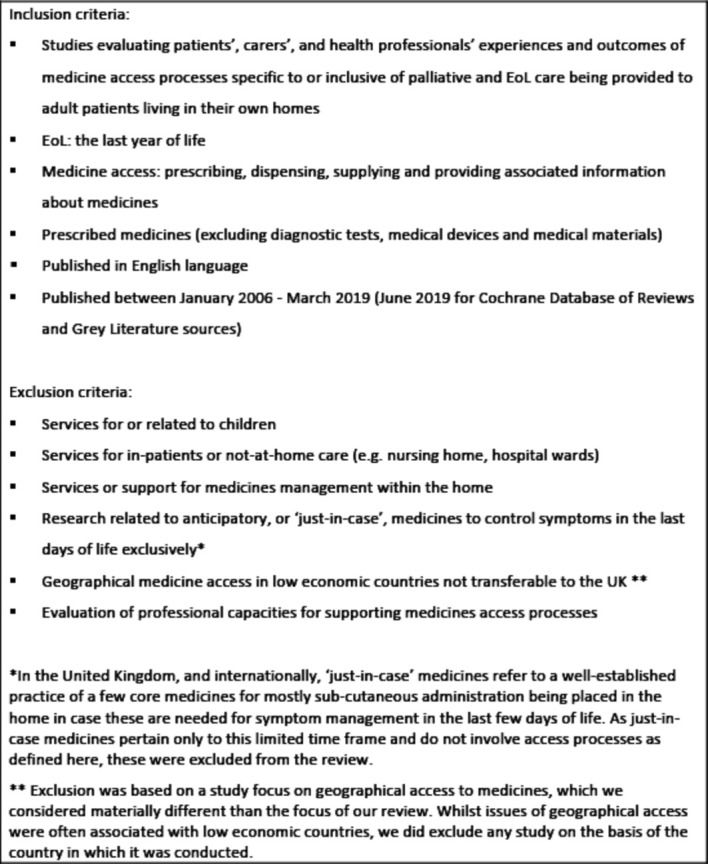
Phase 1 study inclusion and exclusion criteria. EoL, end of life.

Inclusion and exclusion criteria are summarised in figure 1. As described above, medicine access was defined as prescribing, dispensing, supplying and providing associated information about medicines; EoL was defined as the last year of life. The search period start date was 2006 because this was the point at which nurses and pharmacists in the UK gained the legal ability to independently prescribe any medicine from the British National Formulary.^9^ Children and young people under 18 years of age were excluded as our focus was on access to medicines for adults living at home.

#### Selection process

Studies were selected for inclusion using a two-step process: one researcher (MO) screened titles (and abstracts when necessary) to remove duplicates using data management software (EndNote V.X8.2, Clarivate Analytics). Following deduplication, titles and abstracts were screened to determine study eligibility. To ensure the validity of the process, one reviewer (MO) and a second reviewer (NC) each independently screened a random sample of 10% of abstracts; screening results were double-checked by a third reviewer (SL). After resolving disagreements and achieving consensus, the remainder of the screening was undertaken by a single author (MO). Following initial decisions on papers for inclusion, two review authors (MO and SL) each independently reviewed full-text articles against all inclusion and exclusion criteria and resolved disagreements through discussion in order to achieve consensus.

### Data extraction and quality appraisal

Data extraction was conducted for each eligible study by a single reviewer (MO) and checked against the manuscript by another reviewer (SL). Quality appraisal was undertaken using the Mixed Methods Appraisal Tool, which includes tools for different study designs and is therefore appropriate for systematic reviews that include qualitative, quantitative and mixed methods research studies.^10^ Appraisal was undertaken by a single reviewer (MO), recorded on a data extraction sheet and checked by another reviewer (SL). Data management software (EndNote) was used to organise the search results and references.

### Synthesising and interpreting results

The search identified that studies included qualitative, quantitative and mixed methods research. Thus, a framework for a mixed studies review was used, with thematic analysis of qualitative data within a data-based convergent synthesis design.^11^ In this design, qualitative and quantitative data can be analysed and synthesised together. Overlaying this, however, and in keeping with the study focus, we structured the review according to models of service delivery that we found in the literature reviewed: community pharmacy services; general practitioners (GP; family doctors); community specialist nurse independent prescribers; and hospice emergency medication kits (HEMK).

## Results

**Figure 2:**
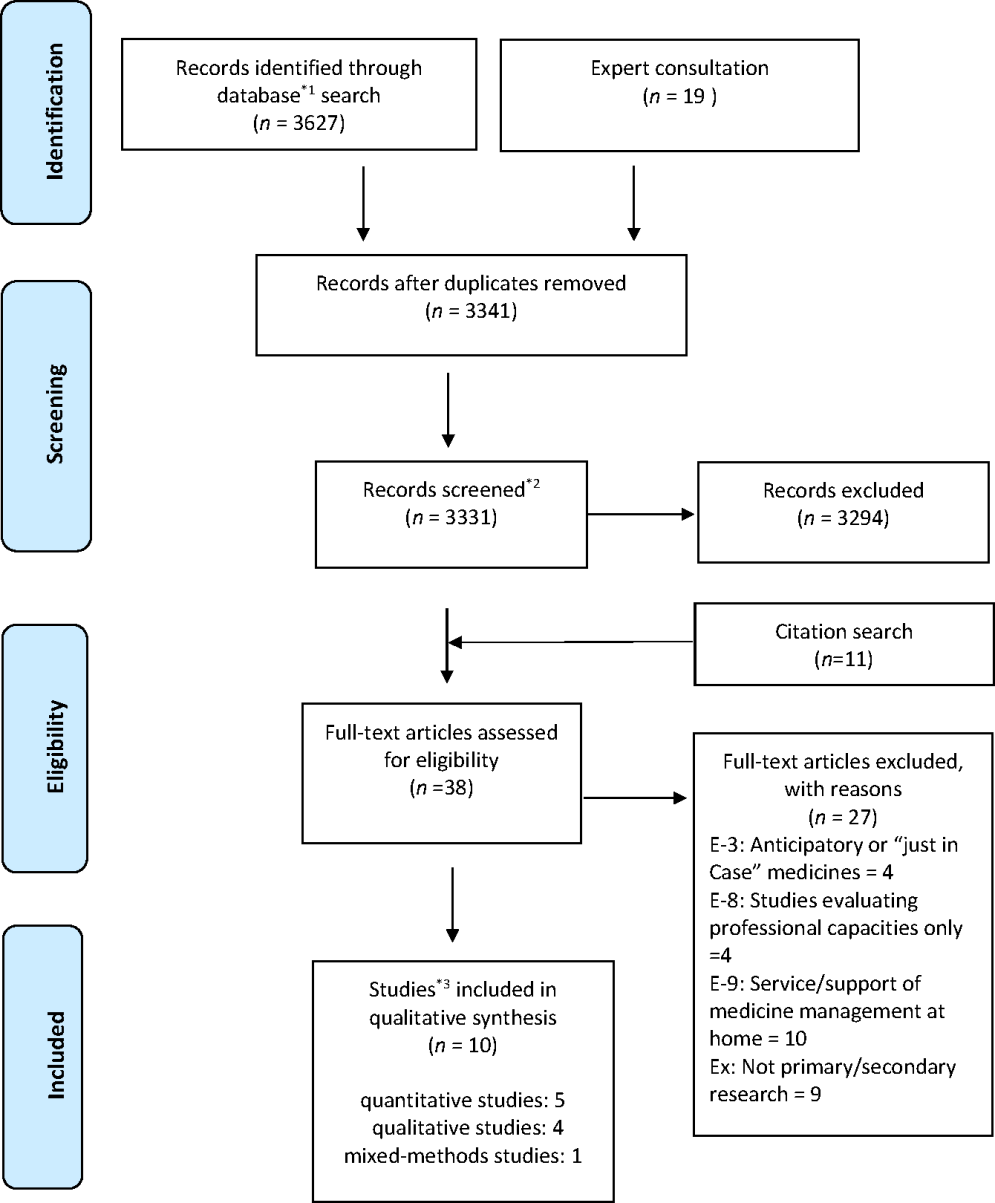
Preferred Reporting Items for Systematic Reviews and Meta-Analyses (PRISMA) 2009 flow diagram. *1Database; MEDLINE: 1306+CINAHL: 167+PsycINFO: 968+EMBASE: 1186. *2The reason for reduction; sources were not accessible: 10. *3Two papers reporting the same study were counted as one study.

The electronic database search produced 3627 records and 19 records were identified by expert consultation, as shown in the PRISMA flow diagram in figure 2. Ten studies were included in the review: five quantitative studies, four qualitative studies and one mixed methods study (online supplemental table 2). One study was reported as a short series over two editions of a journal^12 13^; these were linked together for the purposes of the current review.

All studies were from economically developed countries: the UK, Ireland, the USA, Australia and Japan. The majority of the included studies used small sample sizes and were locality-based studies limited to a few districts or institutions, with the exception of one nationwide study in Japan. In general, the quality of each study was variable in terms of methodological rigour; limitations were that samples were often insufficiently described, making conclusions about generalisability or transferability difficult, the development of data collection tools often lacked detail and surveys had low response rates, making response bias difficult to rule out. There were no randomised controlled trials or large-scale studies (details of quality assessments are shown in online supplemental table 3).

### Medicine access and community pharmacy services

Four studies focused exclusively on investigating medicine access experiences related to community pharmacy,^14^,^17^ of which two studies^15 17^ included pharmacies providing enhanced or commissioned palliative medicine services (in the UK, this typically involves keeping a core stock of palliative medicines and extended opening hours for access). Two further studies included evaluation of community pharmacy medicine access as part of investigating the broader model of community service provision for patients receiving home-based specialist palliative care.^18 19^ Two studies focused exclusively on the perspectives of community pharmacists in providing access^14 15^ while three studies also included patients and other health professionals,^17^,^19^ and Bennie *et al* focused solely on patients’ views of community pharmacy access.^16^

#### The access experience

Overall, findings from all six studies revealed a picture of delays and problems with accessing medicines from community pharmacies; these were focused on pharmacy stock of medicines and on information provision.

##### Pharmacy stock of palliative care medicines

Ise *et al* provide some quantitative data on access, indicating less than comprehensive provision, with 77% of the 1036 community pharmacies in their nationwide survey in Japan holding a ‘narcotics’ (opioid) licence and only 50% reporting involvement in monthly provision of opioids.^14^ It is unclear why the other 23% of pharmacies did not hold a licence or how this affected the patient experience of accessing opioids, but Ise *et al* comment that a system whereby all community pharmacies can supply opioids to all patients who need them has clearly not been established. Certainly, lack of community pharmacy stock of palliative care medicines generally is also reported as problematic in other studies.^15 17 18^ Miller found that although 44/55 (80%) of the patients/carers surveyed reported receiving palliative medicines on their first visit, 10/54 (1 missing data, 19%) had to travel to more than one pharmacy before accessing the medicines and 40/55 (73%) reported that the medicines were ‘needed urgently’.^17^ Although not measured quantitatively, community pharmacists in Akram *et al*’s study of four localities in Scotland also reported delays in being able to provide palliative care medicines, and these pharmacies were part of an enhanced commissioned service and specialist network to provide these medicines to patients in the community.^15^ On the other hand, Lucey *et al*’s study, a systems analysis of obtaining medications for patients under the care of a specialist palliative home care provider, reported that in 54% of 22 patient-reported medicine changes during the study period, medication was received without delay. However, the numbers of patients were small (n=12).^18^ Nurses’ reports in this study also showed only 12 instances of delays across 57 patients over a 12-week period—the majority of these being over 48 hours’ wait.^18^ The main reason for delays was due to no stock in the community pharmacy.^18^ The overall number of medicine access episodes that nurses reported on is not clear, but 12 delays over 12 weeks for 57 patients seem a relatively small number of delays.^18^

##### Information provision

Data on community pharmacy information provision for patients are available in three studies,^14 16 19^ and again show there is scope for improvement. Patients and carers in Bennie *et al*’s focus groups saw the pharmacist as a source of medicine information (as well as GPs) if they had a relationship with them. However, some reported little contact with a community pharmacist and overall knowledge of services offered was poor, with patients often acquiring knowledge in an unplanned way through family/friends or when in a crisis situation.^16^ Patients and carers wanted a more proactive role from pharmacists, in particular regarding prescription and supply processes of controlled drugs or when a new medicine was prescribed.^16^ The sample in this study was small: 14 patients and 13 carers from one city in Scotland.^16^ Nevertheless, findings from Ise *et al*’s national survey also highlight gaps in information provision: 50% of the 1036 pharmacists in their study reported that they did not counsel patients about their palliative care medicines, primarily because they lacked information about the patient (eg, disease status and awareness of illness and opioids), and less than 5% had a room to counsel the patient privately.^14^ In Australia, carers and patients felt that sometimes the information provided was inadequate for them to manage their medications at home appropriately.^19^

### Influences on accessing medicines from community pharmacies

#### Communication, collaboration and education

Four studies report on influences that challenge or support the access process.^14 15 17 18^ Many of these are concerned with communication between the pharmacist and other members of the healthcare team. Communication with the pharmacy was reported as problematic, causing delays in supplying medicines, through a mismatch between prescriptions received and stocks held^15^ and/or a lack of information on the palliative care status of the patient.^14 15 17^ The latter was either due to the pharmacist seeing unfamiliar patients who had been referred by another network pharmacist^15^ or health professionals’ reluctance to share information.^17^ A finding reported by both studies that included enhanced or commissioned services also related to communication^15 17^: medicine access was adversely affected by health professionals’ lack of knowledge about these services. Miller concludes that ‘*poor healthcare professional (HCP) knowledge of which pharmacies stock palliative medicines meant patients and their families were not always able to access medicines promptly*’ and that HCPs need to routinely be made aware of such services and their locations.^17^ Similarly, patients and carers in Bennie *et al*’s study also reported a lack of awareness of more general pharmacy services. Pharmacists in Akram *et al*’s study also reported that communication was disrupted when patients transferred between secondary and primary care settings, affecting prescriptions and the ability to swiftly supply medicines needed.^15^

Little data were available on factors supporting good access, related to the fact that studies highlighted poor access experiences. However, pharmacists in one study stated that pre-emptive communication from community nurses about medicines likely to be required by patients was helpful, as well as being part of a network of pharmacists, where medicines and advice could be accessed.^15^

Akram *et al* also found community pharmacists perceive better training of counter staff and of locum pharmacists is needed, as well as resources for pharmacists to support clinical practice.^15^ Additionally, Miller’s focus group interview highlighted that community pharmacists often have limited experience and knowledge about palliative care medicines.^17^ Furthermore, Kuruvilla *et al*’s findings indicated GPs unfamiliar with palliative care medicine needed support, and a palliative care specialist pharmacist could be valuable for such GPs and be an integral role for a community palliative care service.^19^

#### Practical problems with palliative medicine stock and couriering medicines

Miller found pharmacists reported practical difficulties keeping palliative medicines in stock—for example, secure storage space and the wide range of opioid dosage requirements for individual patients.^17^ Seventy per cent of pharmacists in Ise *et al*’s survey also said that being able to have a swift supply from, and ability to return opioids to, wholesalers would be useful, as well as being able to get stock from other local pharmacies.^14^ Further obstacles were also identified: 33% (19) of pharmacists completing questionnaires in Lucey *et al*’s study reported difficulties in accessing stock and 49% that medicines not being on state reimbursement schemes caused delays.^18^

In addition, the authors report the other main factor causing delays was no one to courier prescriptions/medication to and from GP, pharmacy and patient. The difficulty of picking up medications from community pharmacy by patients or carers was also noted by Miller.^17^

In another study, families’ involvement in collecting prescription or medicines and delivery services seemed to be helpful, though the latter was not always available and they were a financial burden for some patients when delivery was not free of charge.^19^ Additionally, patients and families receiving medicines in this way seldom interacted directly with pharmacists.^19^

### Outcomes of community pharmacy medicine access

There are little data on outcomes reported in the studies reviewed. One study reported that commissioned service pharmacies stocking an agreed list of palliative care medicines could shorten the time required to obtain urgently needed palliative medication compared with medication being provided by a non-commissioned service pharmacy.^17^ The median time taken for accessing urgent palliative care medicines was significantly longer for non-commissioned service pharmacies (5 hours) compared with pharmacies commissioned to hold stock of palliative care medicines (10 min, p=0.002).^17^ Additionally, compiling tailored lists of palliative care medications through communication between pharmacies and GPs resulted in a similar time saving in commissioned pharmacies.^17^ However, the impact of this on outcomes such as symptom control, patient and carer distress or use of emergency health services was not evaluated.

### GPs (family doctors)

Only one study included data on GPs’ experiences of providing medicine access, focusing on delays and the causes of these.^18^ Questionnaires were sent to 268 GPs in one city, asking them to select the most common causes of delay from a prespecified list.^18^ One hundred and eleven questionnaires were returned (41% response rate).^18^ No delay was reported by 34% of GPs.^18^ The most commonly cited factor causing delay was the need to clarify the advice given by the home care team (30.6%), followed by the inability of someone to collect the prescription (23.4%) and 18.9% of respondents reported the patient being unable to attend the surgery as a cause of delay.^18^

### Community specialist nurse independent prescribers

One study conducted interviews with six independent nurse prescribers employed as community palliative care clinical nurse specialists in an interpretive phenomenological study.^12 13^ The study was conducted in one region in England and aimed to understand the lived experience of these nurses prescribing for palliative care patients in the community.^12^ They found the most significant perceived benefit of nurse prescribing, reported by all six nurses, was that it enabled patients to access medication quickly, particularly near the end of their life, leading to effective symptom management.^13^ The majority of the nurses also considered that it was during out of hours that the ability to prescribe independently had the most impact, preventing delays by avoiding the need to call an out-of-hours doctor, which could reportedly take many hours.^13^ The authors conclude: ‘*The ability of community palliative care clinical nurse specialists to prescribe can facilitate rapid access to medicines, particularly during out-of-hours periods*’^13^ (p 133).

### Hospice emergency medication kits

Three studies from the USA evaluated the outcome of HEMKs regarding use, impact and cost.^20^,^22^ HEMKs are typically ordered by a physician on referral to home hospice service, and kept in the patient’s home, to allow the patient access to small quantities of medication that can be administered immediately on nurse instruction.^20^,^22^ Emergency medication kits contain sufficient medications for 12–72 hours, thus avoiding the immediate need for pharmacy and physician involvement after hours.^21^ (HEMKs were intended for use in any emergency across an extended period at EoL and contained a wide range of medicines, including, for example, antibiotics. We therefore considered these studies met our inclusion criteria, and were not equivalent to ‘just-in-case’ boxes, which were excluded from the review.) All studies included an evaluation of the perceived impact of HEMKs, which shed light on their effect on access to medicines. In all studies, clinicians providing care were asked about HEMKs’ impact on unplanned healthcare resource use; the majority considered that kits averted use of other services. For example, 93% of the 78 home hospice nurses completing a questionnaire survey in one study reported that an emergency department visit or hospitalisation was avoided by having a kit in the home, with 26.1% reporting this was ‘often’ and 40.6% ‘very often’.^22^ Clinician views on helpfulness and patient satisfaction were also positive across the two studies measuring this, with 59% of nurses considering HEMKs to be helpful 100% of the time^22^; and 100% (n=13) of the hospices using HEMKs in the other study reporting it increased both patient and nurse satisfaction.^21^ In a comparison between a hospice using HEMKs for some patients and one not using them at all, Walker and McPherson also report the after-hours nurses perceived caller (patient/family) satisfaction was significantly higher in hospice patients with a kit compared with both the non-kit hospice patients and the HEMK hospice patients without a kit (95%, 75% and 82%, respectively; p<0.001).^21^ One study also measured the impact of HEMKs on perceived symptom relief time.^21^ Nurses in the hospice using HEMKs estimated 56% of after-hours callers received symptom relief within 30 min, whereas nurses reported none of their callers from the hospice without kits were treated satisfactorily in less than 30 min.^21^

Overall, while HEMKs were found to have a positive impact on a number of medicine access indicators in all three studies—including, perhaps, notably shorter time to symptom relief and reduced use of emergency services—all were small scale. Two studies were confined to a state-wide hospice survey,^20 21^ and one focused on a sample from one medical centre only, resulting in overall small numbers of hospices, clinicians and patient records included.^22^ In addition, the majority of the data from all studies were limited to clinicians’ perceptions only, with limited objective data and no patient or carer experiences captured.

## Discussion

This review found sparse research in this area: 9 of the 10 studies were small-scale or pilot studies and local samples. Although it is difficult to generalise the results, this review identified several problems with current models and also highlighted potential approaches to improving medicine access.

Overall findings suggest there are problems with accessing medicines via community pharmacies, and a number of issues delaying access have been identified, many of which focus on either pharmacy stock or communication between the pharmacy and health professionals, and with patients. However, with the exception of one study (Ise *et al*), sample sizes were small and pertain to only a few localities, and there is a lack of data on outcomes of access experiences.

Wider literature also reports deficiencies in community pharmacy stock of palliative medicines,^23^ and in our review we found pharmacies commissioned to provide stocks of locally agreed palliative medication lists reduced delays in medicine access and tailored lists produced through communication between pharmacies and GPs worked similarly.^17^ However, this study evaluated only one local service and city area; thus, evaluation of these services on a wider scale is warranted. Alternatively, improvements upstream in the supply chain to community pharmacies might also be effective in avoiding potential delays—further research into the effectiveness of the supply chain of palliative care medicines is also required. Studies also suggested a number of problematic issues were linked to communication between pharmacists and patients (patients’ lack of awareness of services, and of opportunity for information provision about medicines) and/or between pharmacists and other health professionals (pharmacists’ lack of awareness of patients’ palliative care status, health professionals’ lack of awareness of commissioned or enhanced pharmacy services). Patients and carers’ needs for information about medicines in this context have been repeatedly identified,^24^,^26^ and the potential role of the pharmacist in fulfilling such needs also highlighted. For example, Latif *et al* recommend that pharmacists should elicit patient’s level of understanding,^27^ their concerns about medicines, and provide tailored information to ensure medicine optimisation. The review reported here suggests more proactive awareness raising of pharmacy services, and more pharmacist engagement in information giving continues to be required. To address the communication gap between community pharmacy and the wider healthcare team, greater integration of the pharmacist into the primary healthcare team would be advantageous. Calls for such actions for the pharmacy profession have been made in other contexts.^28^ Our review also suggests there may be a case for improved training and education or support for community pharmacists and GPs as well as counter staff and locum pharmacists, a recommendation made elsewhere in relation to generalists and specialist palliative care medicines.^29 30^ Further to the problems identified in this area in their 2012 study, Akram *et al* also reported a promising initiative of a specialist palliative care pharmacist who provided education for, and facilitated involvement of, community pharmacists in palliative care locally.^31^ This kind of approach might assist collaboration among community HCPs and support better information provision; the model of a specialist palliative care community pharmacist has recently been recommended in national policy in this area.^32^ Wider examination of this area is warranted.

Three studies in this review suggested that HEMKs—kits comprised a number of palliative care medicines stored in the patient’s home that could be used in an emergency—can avert hospital admissions and emergency department visits and improve quality of care at home by providing timely access during out of hours. Accessing services and medicines out of hours is known to be particularly problematic and a focus for service delivery recommendations,^33^ and so the use of such kits in the home offers a promising way to deal with this. A qualitative evaluation of HEMKs introduced in an Australian setting^34^ adds to the positive data on this form of service delivery: caregivers ‘overwhelmingly’ (p 486) viewed the introduction of the kit into the home as positive, citing accessibility, timeliness and symptom control as benefits. The study also reported some carers lacked confidence and expressed concerns about administration of medicines in the home, an issue which the studies in the review reported here did not explore. Although a more limited form of ‘just-in-case’ medicine kits is used in the UK in the last few days of life, more data are needed to consider the applicability of HEMKs in the context of different healthcare systems.

Couriering of medicines and the ability of palliative care patients and carers to be involved in collecting prescriptions or medicines were raised in two studies.^17 18^ Solutions such as electronic prescribing and transfer to the pharmacy (in the UK, a system which now includes prescription of controlled drugs) and home delivery may be of value to overcome this. But one study (Kuruvilla *et al*)^19^ highlighted that delivery systems are not free of charge and reduce contact time with the pharmacist, and a more recent survey highlighted electronic prescribing is far from universally available to prescribing nurses and pharmacists, suggesting further in-depth evaluation of these aspects of service delivery is required.^35^

Community palliative care specialist nurse prescribers were also reported to provide out-of-hours support and quicker medicine access in times of crisis. This form of out-of-hours care delivery is also recommended as a quality improvement priority by Williams *et al*^36^ following their review of community palliative care incidents reported on a national database. However, evaluative data in the review reported here were confined to one small-scale study and therefore further evidence, including larger scale studies and insights of other stakeholders, is needed to endorse the value of nurse and pharmacist prescribing in this context.

The review also highlights that a variety of indicators have been used to measure medicine ‘access’, including patient-reported and nurse-reported length of time to receive medicines, delay/no delay experienced, number of pharmacies visited, stock held by pharmacies, supply chain issues, whether information on medicines is provided, as well as characterisation of a range of influences on access and supporting access to medicines. Drawing these systematically together may be useful in informing the design of future research and service evaluations of interventions to promote better access to medicines in the community setting.

Any overall conclusions of the review are limited by the quantity and quality of the research included in the review. Only a few models were studied, and only one study used a systems approach,^18^ studying different components of service delivery as a whole—all other studies focused on one component only, which is not representative of how patients experience care and the process of accessing medicines—from prescription request through to dispensing, supply and information giving about medicines in the home. Medicine access provision by professionals such as generalist community nurses and family doctors is understudied, as well as more recent initiatives such as nurse, pharmacist and paramedic prescribing, and developments such as telephone support lines available out of hours. Most studies in the review were small scale and many only included access as part of a broader focus, thereby further reducing the available data. Some models of care delivery—for example, hospice emergency kits and nurse prescribing—will vary between countries and therefore the transferability of findings across international systems of care delivery is also limited. Most studies focused on health professional self-report data and only four studies included views of patients.^16^,^19^ There were very little data on outcomes of medicine access experiences and so comparison between models was not possible.

### Strengths and limitations

To our knowledge, the review is the first to systematically analyse international studies on the important issue of experiences of accessing palliative care medicines in the community. The review included a comprehensive search of grey literature sources, as well as consultation with experts to ensure unpublished literature was identified.

As the review was part of a larger study focused on medicine access within different models of community palliative care provision, we had an a priori focus on service delivery models that determined the structure of our review. The focus on models of care provision makes extrapolation of findings to different international contexts difficult; countries differ, for example, in the prescriptive authority afforded to nurses and pharmacists and the extent to which electronic prescribing is available for health professional prescribers to use.

We did not exclude studies on the basis of quality assessment, and the review is limited by the quantity and quality of research in this area (see above). Studies used heterogeneous indicators to measures experiences and outcomes of medicine access—therefore, comparisons between studies and models were not possible.

## Conclusions

Despite suggestions that accessing palliative care medicines to manage symptoms at home is problematic for patients, there is very little large-scale or in-depth research into these experiences and how models of service delivery influence access and subsequent clinical outcomes and health service use. Further research evaluating both established and more recent service delivery models is required, which includes patient and carer perspectives and the measurement of outcomes.

## supplementary material

10.1136/bmjspcare-2020-002761 online supplemental file 1

10.1136/bmjspcare-2020-002761 online supplemental file 2

10.1136/bmjspcare-2020-002761 online supplemental file 3
