# Supplementary material for: Community access to palliative care medicines – patient and professional experience: systematic review and narrative synthesis
Source: BMJ Support Palliat Care. 2021 Mar 28;14(e2):e002761. doi: 10.1136/bmjspcare-2020-002761 (PMC11671949; doi:10.1136/bmjspcare-2020-002761)
Supplement: online supplemental file 1 [file bmjspcare-14-e2-s001.pdf]

Online supplemental material Table 1. Search terms for MEDILINE(EBSCO)

S1	(MH "Palliative Care")
S2	(MH "Terminal Care")
S3	palliative n3 care
S4	terminal N3 care
S5	"end of life" or EOL
S6	palliat*
S7	terminal and patients
S8	medicat*
S9	medicine*
S10	drug*
S11	(MH "Pharmacies")
S12	(MH "Community Pharmacy Services")
S13	access*
S14	suppl*
S15	dispens*
S16	distribut*
S17	availab*
S18	deliver*
S19	administ*
S20	"out of hours"
S21	prescri*
S22	pharmac*
S23	primary N3 care
S24	primary health care
S25	home
S26	communit*
S27	general practice
S28	(MH "General Practice")
S29	"Family practice"
S30	local N3 Pharmac*
S31	"Community practice"
S32	Community N3 Pharmac*
S33	S1 OR S2 OR S3 OR S4 OR S5 OR S6 OR S7
S34	S8 OR S9 OR S10
S35	S11 OR S12 OR S13 OR S14 OR S15 OR S16 OR S17 OR S18 OR S19 OR S20 OR S21 OR S22
S36	S23 OR S24 OR S25 OR S26 OR S27 OR S28 OR S29 OR S30 OR S31 OR S32
S37	S33 AND S34 AND S35 AND S36<br>Limiters - Date of Publication: 20060101-20180331; English Language; Human; Age Related: All Adult: 19+ years

MH: medical subject headings, N3: allowing 0 to 3 words between the words
