# Supplementary material for: Community access to palliative care medicines – patient and professional experience: systematic review and narrative synthesis
Source: BMJ Support Palliat Care. 2021 Mar 28;14(e2):e002761. doi: 10.1136/bmjspcare-2020-002761 (PMC11671949; doi:10.1136/bmjspcare-2020-002761)
Supplement: online supplemental file 2 [file bmjspcare-14-e2-s002.pdf]

Online supplemental material Table 2 Details of included studies

Reference, country	Model/s of service delivery	Study Aim and Design	Participants and sample size	Results and findings related to access
Lucey et al. 2008 Ireland	Specialist palliative home care, including community pharmacy, General Practice and specialist home care nursing.	A systems analysis to highlight factors causing delays in the process of obtaining medications for patients under the care of a specialist palliative home care provider<br><br>Quantitative Questionnaires and structured 'observation' data	1) Survey<br>111 GPs registered in North Dublin<br>57 Retail pharmacists registered in North Dublin<br>22 patients who were referred to one hospice home care team<br><br>2) Observational cohort study (12 reports by nurses in specialist care team)	1) Perceived factors causing delay in medicine access process:<br>**GPs**: need to clarify the advice given by the home care team (30.6%); the inability of someone to collect the prescription (23.4%); the patient being unable to attend the surgery (18.9%)<br>**Pharmacists**: some specialist palliative care medications were not stocked (49.1%), difficulty in obtaining the supply of these types of medications (33%); medication not being available on the medical reimbursement system (GMS) (49%)<br>**Patient results**: received their medication without delay (54%); had not been advised to change their medication by the home care team (46%)<br>2) Nurse reported 12 delays > 24 hours over a 12 week period; 11 delays were > 48 hours, and 6 delays were due to medication not being in stock.
Bishop et al. 2009 USA	Hospice emergency medication kit (HEMK)	To survey all active hospice programs about their use of HEMKs<br><br>Quantitative Telephone survey	22 Clinical nurse directors or nurse managers in one state	86% of agencies reported HEMK often averted hospital or emergency department visits.
Walker and McPherson 2010 USA	Hospice emergency medication kit (HEMK)	To compare perceptions of hospice managers and clinicians regarding the value of Emergency Medication Kit (HEMK) and to assess outcomes<br><br>Quantitative Surveys	1) 21 Clinical directors (or equivalent) at hospices in one state<br><br>2) Hospice A (with HEMK) = 154 patients<br>Hospice B (without HEMK) = 103 patients	1) 14 of 21 hospices reported using HEM.<br>Perceived value of HEMK were preventing emergency department visits (69%), unscheduled nursing visits (54%) and pharmacy deliveries (77%) as well as increased satisfaction of both patients and nurses (both 100%).<br>2) Estimated time to symptom relief was shorter in Hospice A (with kits) patients than in Hospice B; Hospice A =56% received symptom relief within 30 minutes but Hospice B: none were satisfactorily treated in the same duration.

				After-hours nurses perceived caller satisfaction was significantly higher in Hospice A patients with a HEMK compared to both Hospice B and Hospice A patients without a HEMK.
Ise et al.<br>2010<br>Japan	Community pharmacy	Quantitative<br>Questionnaire survey<br><br>To evaluate;<br>(1) the availability of and the experience of pharmacists providing narcotics (opioids) through community pharmacies<br>(2) availability of patient counselling by pharmacists;<br>(3) perceived difficulties in treating cancer patients with opioids;<br>(4) useful strategies to make opioids more available to patients	1036 community pharmacies as a representative national sample, (response rate: 34.5%).	77% of community pharmacies had a narcotics retailer license, 50% received prescriptions for and prepared narcotics each month.<br>Half of the pharmacists reported that they did not counsel patients, primarily because they lacked information about the patient<br>Pharmacists reported communicating with terminally ill cancer patients was extremely difficult.<br>76% of community pharmacists felt it was important to be able to return opioids to wholesalers to make opioids more easily available to patients
Akram et al.<br>2012<br>UK	Community pharmacy	Understand the provision of enhanced service community pharmacies (Community Pharmacy Palliative Care Network pharmacies offered specialised palliative medicines stock, urgent medicines delivery service and support to other health care professionals)<br><br>Qualitative<br>Focus group interviews	35 pharmacists in 5 focus groups	Barriers for pharmacists providing palliative care medicines in community pharmacy:<br>1) Medication supply stock issue; medicines not listed; out-of-hours prescriptions; prescribing error with controlled drugs; infrequent use of delivery service provided by other staff<br>2) Communication<br>▪ Communication issues across the palliative care team (e.g. necessity of contacting pharmacy in advance to prevent supply problems, poor communication during transition of hospital admission/discharge)<br>▪ Membership in the CPPC Network was seen as supportive but also meant unfamiliar patients were referred to them<br>3) Education and training

				▪ Limited understanding of the role by other PC team members<br>▪ Better training for counter assistants and locum pharmacist<br>Availability of routine resource for pharmacists to support clinical practice (e.g. web-page)
Bennie et al. 2013	Community pharmacy	To investigate how older palliative care patients and their carers access information about their medication and what knowledge they have of community pharmacy services<br><br>Qualitative<br>Focus group interviews	14 patients (aged 65–79)<br>13 carers (aged 57–74) took part in six separate focus groups.	1) Access to medication information<br>▪ Patient information leaflets appeared to be most common source of information about drugs<br>▪ Participants preferred face-to-face interactions when possible<br>2) Study participants had limited knowledge of available help for them from their community pharmacist regarding their medications; a more proactive role of community pharmacists in disseminating relevant and tailored information to older palliative care patients was desired.
Leigh et al. 2013<br>USA	Hospice emergency medication kits (HEMK)	To evaluate Hospice Emergency Kit (HEK), medication utilization, safety, diversion and perceived impact<br><br>Quantitative<br>Chart abstraction and questionnaire survey	76 deceased veterans discharged to home hospice<br><br>78 hospice nurses in 16 community hospice agencies (49% response rate)	59% of the nurses felt HEMKs were helpful 100% of the time (mean 84.2%) and 93% felt an emergency visit or hospitalization was avoided by having the kit in the home.
Miller 2017<br>UK	Community pharmacy	To investigate the delivery of palliative care services by community pharmacists in Sheffield<br><br>Mixed-methods<br>Prescription analysis; customer survey; health care professional interviews	1) 5 community pharmacies<br>2) 75 prescription data collection forms<br>3) 55 customers<br>4) Pharmacists: 6<br>A) Community pharmacists who participated in Phase 1 = 5<br>B) Working in intermediate care = 1<br>5) Doctors: 4	1) Stock availability led to delays: approximately 1 in 5 customers had to go to more than one pharmacy to get urgently required palliative care medication, with one in two customers who were referred by a healthcare professional needing to go to more than one pharmacy.<br>2) No evidence that errors on prescriptions led to a delay in obtaining palliative medication.<br>3) The median time taken for accessing urgent palliative care medicines was significantly longer for non-commissioned service pharmacies (5 hours) compared to pharmacies commissioned to hold stock of palliative

			A) GP = 3<br>B) Specialist palliative care doctor = 1<br>4) Nurses: 6<br>A) District/community = 5<br>B) Specialist palliative care nurse = 1	care medicines (10 minutes p=0.002)<br>4) Pharmacies drew up their own local list of palliative care medications that worked with local GP surgeries to keep in stock and had similar minimum times for accessing medication as commissioned service pharmacies.<br><br>Factors facilitating or limiting medicine access<br>1) Skills, knowledge and training for anticipating needs<br>2) Collaborative relationships for planning ahead for medicine stock.
Kuruvilla et al. 2018<br>Australia	Community palliative care services	To explore the perspective of stakeholders about gaps in the current model of community palliative care services and assess opinion on the role of a specialist palliative care pharmacist<br><br>Qualitative<br>Focus group interviews	Healthcare professionals (n=12):<br>3 Palliative care medical consultants<br>1 Palliative care medical-registrar<br>1 GP<br>1 Palliative care nurse-consultant<br>1 Palliative care nurse-practitioner<br>2 Community nurses<br>1 Hospital pharmacist<br>1 Community pharmacist<br>Palliative care patients/ caregivers (n=8)	Themes from the focus groups:<br>• Gaps in access to medicines/resources / information:<br>Lack of access to pharmacy stock, medicines information, and patient medical records, especially outside business hours and difficulties for patients living in rural and remote areas were particular barriers highlighted by focus group participants.
Latham and Nyatanga 2018<br>UK	Community specialist nurse independent prescribers	To explore the lived experience of clinical nurse specialists working as independent prescribers<br><br>Qualitative<br>Interviews	6 Community specialist nurse non-medical independent prescribers	1) Perceived benefits of nurse independent prescribing:<br>A) Helping patients in times of crisis<br>B) Being available when others are not (out-of-hours)<br>2) Barriers to prescribing practice:<br>A) Anxiety about writing prescriptions incorrectly<br>B) Inadequate knowledge and negative/unfavourable attitudes of other HCPs<br>C) Inadequate access to patients’ medical information
