# Supplementary material for: Community access to palliative care medicines – patient and professional experience: systematic review and narrative synthesis
Source: BMJ Support Palliat Care. 2021 Mar 28;14(e2):e002761. doi: 10.1136/bmjspcare-2020-002761 (PMC11671949; doi:10.1136/bmjspcare-2020-002761)
Supplement: online supplemental file 3 [file bmjspcare-14-e2-s003.pdf]

Online supplemental material Table 3. Quality assessments of included studies.

Qualitative studies	1.1. Is the qualitative approach appropriate to answer the research question?	1.2. Are the qualitative data collection methods adequate to address the research question?	1.3. Are the findings adequately derived from the data?	1.4. Is the interpretation of results sufficiently substantiated by data?	1.5. Is there coherence between qualitative data sources, collection, analysis and interpretation?
Akram et al. 2012	Yes	Yes<br>35 pharmacists in 5 focus groups<br>Verbatim transcription	Yes<br>Framework Analysis<br>Independent coding and validation process	Yes<br>Quotation is adequately employed for each theme.	Yes
Bennie et al. 2013	Yes	Can't Tell<br>No description of the topic guide.	Can't Tell<br>No description of the method of qualitative analysis	Yes<br>Quotation is adequately employed for each theme.	Yes
Kuruvilla et al. 2018	Yes	Yes	Yes<br>Framework Analysis<br>Independent coding and discussion of discrepancies	Yes	Yes
Latham and Nyatanga 2018	Yes	Can't Tell<br>Full details of purposive sampling not given.<br>Interview schedule validation unclear.	Yes<br>Constant comparative method is reasonable for interpretative phenomenology.	Yes<br>Quotation is adequately employed for each theme.	Yes

Quantitative studies	4.1. Is the sampling strategy relevant to address the research question?	4.2. Is the sample representative of the target population?	4.3. Are the measurements appropriate?	4.4. Is the risk of nonresponse bias low?	4.5. Is the statistical analysis appropriate to answer the research question?

Lucey et al 2008	Yes<br>All GPs, pharmacists in one city area; all patients over a 3 month period in the hospice; unclear details of if / how nurses were sampled.	Can’t Tell<br>Details of respondents are not given, other than as part of the target sample	Can’t Tell<br>Details of the development of the questionnaires are not given	No<br>Response rates were: GPs 41%; pharmacists 33% and patients 38.5%.	Yes
Bishop et al. 2009	Yes<br>Sampling process is adequate; surveyed all active programs in the target area	Can’t Tell<br>Clear description of the sample and reasonable as a local study but difficult to understand if generalisable across country or globally.	Yes<br>The survey question was reviewed by a third party and seems to be reasonable.<br>Data collection method of telephone interview was not described.	Yes<br>All candidates were contacted and response rate of the survey was 22/22.<br>Results from 1 program were not included due to limited response.	Yes<br>Descriptive statistics
Walker and McPherson. 2010	Can’t Tell<br>1) Sampling process for the survey is adequate; surveyed all active programs in the target area.<br>2) No details of the method of sampling hospices or nurses within them for the comparative study.	Can’t Tell<br>1) Details of survey respondent sample and hospices are lacking.<br>2) No details of hospices or nurses taking part in the comparative study are given.	Yes<br>1) Survey questions seem to be reasonable though it was only internally reviewed. Data collection method of telephone interview was not described.<br>2) Methods for comparing frequency, estimated cost and client satisfaction were reasonable.	Yes<br>1) 21/23 programs in the area participated and 14 reported using EMK<br>2) No details are given on response rates of nurses in the hospices	Yes<br>Descriptive statistics
Ise et al. 2010	Yes<br>Random sampling from a community pharmacist database	Yes<br>Clear description of the sample with adequate methods.	Yes<br>The survey question was clearly defined and seemed to be reasonable though it was only internally reviewed.	No<br>Response rate was low at 34.5%.	Yes

Leigh et al. 2013	Can’t Tell<br>No details of sampling method of hospice agencies for nurses	No<br>Only 77% had cared for a veteran with the institution’s HEMK<br>Reasons why eligible individuals chose not to participate were not described.	Can’t Tell<br>No details of the development of the nurse questionnaire	No<br>Response rate of the questionnaire was 49% (78/160); authors acknowledge there may have been a positive response bias	Yes

**Mixed-method study**	**5.1. Is there an adequate rationale for using a mixed method design to address the research question?**	**5.2. Are the different components of the study effectively integrated to answer the research question?**	**5.3. Are the outputs of the integration of qualitative and quantitative components adequately interpreted?**	**5.4. Are divergences and inconsistencies between quantitative and qualitative results adequately addressed?**	**5.5. Do the different components of the study adhere to the quality criteria of each tradition of the methods involved?**
Miller 2017	Yes	Yes	Yes	Can't tell<br>Divergences and inconsistencies were not described in much detail.	Yes<br>1) QUAN (Observational study): The sampling strategy was reasonable but limited participants.<br>2) QUAL (interviews): There was a clear description and reasonable methods.
